# An information gain-based approach for evaluating protein structure models

**DOI:** 10.1016/j.csbj.2020.08.013

**Published:** 2020-08-18

**Authors:** Guillaume Postic, Nathalie Janel, Pierre Tufféry, Gautier Moroy

**Affiliations:** a Université de Paris, BFA, UMR 8251, CNRS, ERL U1133, Inserm, F-75013 Paris, France; b Université de Paris, BFA, UMR 8251, CNRS, F-75013 Paris, France; c Institut Français de Bioinformatique (IFB), UMS 3601-CNRS, Université Paris-Saclay, Orsay, France; d Ressource Parisienne en Bioinformatique Structurale (RPBS), Paris, France

**Keywords:** Protein structure prediction, Model quality assessment, Knowledge-based scoring functions, Statistical potentials

## Abstract

For three decades now, knowledge-based scoring functions that operate through the “potential of mean force” (PMF) approach have continuously proven useful for studying protein structures. Although these statistical potentials are not to be confused with their physics-based counterparts of the same name—*i.e.* PMFs obtained by molecular dynamics simulations—their particular success in assessing the native-like character of protein structure predictions has lead authors to consider the computed scores as approximations of the free energy. However, this physical justification is a matter of controversy since the beginning. Alternative interpretations based on Bayes’ theorem have been proposed, but the misleading formalism that invokes the inverse Boltzmann law remains recurrent in the literature. In this article, we present a conceptually new method for ranking protein structure models by quality, which is (i) independent of any physics-based explanation and (ii) relevant to statistics and to a general definition of information gain. The theoretical development described in this study provides new insights into how statistical PMFs work, in comparison with our approach. To prove the concept, we have built interatomic distance-dependent scoring functions, based on the former and new equations, and compared their performance on an independent benchmark of 60,000 protein structures. The results demonstrate that our new formalism outperforms statistical PMFs in evaluating the quality of protein structural decoys. Therefore, this original type of score offers a possibility to improve the success of statistical PMFs in the various fields of structural biology where they are applied. The open-source code is available for download at https://gitlab.rpbs.univ-paris-diderot.fr/src/ig-score.

## Highlights

• This article describes a new information-theoretic view of statistical potentials.
• We present a new formalism that is conceptually independent of statistical mechanics.
• Its practical validity is shown by the quality assessment of protein structures.

## Graphical abstract

**Figure:**
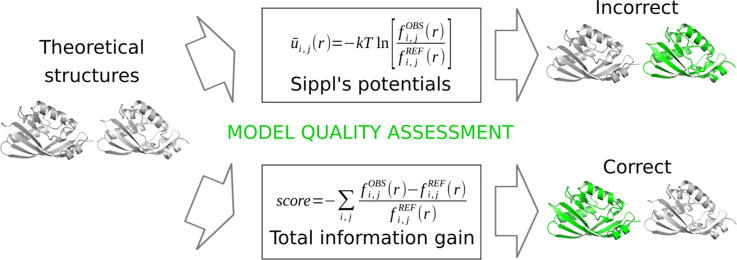

## 1 Introduction

Predicting the three-dimensional structure of a protein is only useful if the model produced is close enough to the native conformation of the macromolecule. According to Anfinsen’s hypothesis, the latter is assumed to be the one with the lowest free energy, in the native conditions [1]. Therefore, being able to discriminate the best model among a set of predicted protein structures requires a scoring function that would behave like free energy, *i.e.* a scoring function whose global minimum would correspond to the native conformation. Free energy estimation may be achieved by generating ensembles of protein conformations, from which the lowest free energy structure can be calculated by using physics-inspired molecular force fields. However, such conformational sampling is computationally costly, which makes these physics-based methods only applicable to a few proteins at a time. Three decades ago, a faster approach has been proposed by M. J. Sippl [2], which consists in constructing scoring functions from interatomic distance (*r*) distributions observed in a dataset of experimentally determined protein structures, as:

(1) $${\bar{u}}_{i,j}\left(r\right)=-kT\text{In}\left[\frac{f_{i,j}^{\mathrm{OBS}}\left(r\right)}{f_{i,j}^{\mathrm{REF}}\left(r\right)}\right]$$

where *ū_i,j_*(*r*) is the estimated free energy of interaction between atoms *i* and *j*, *f_i,j_*^OBS^(*r*) is the observed probability (*i.e.* frequency) of the atoms *i* and *j* being separated by a distance *r* (discretized into bins), *f_i,j_*^REF^(*r*) is a reference frequency aimed at eliminating the sampling bias, *k* the Boltzmann constant, and *T* the temperature. The pseudo-energy of the whole protein is thus computed by summing the *ū_i,j_*(*r*) of every pairwise distance observed in the structure. In this article, for the thirtieth anniversary of this knowledge-based approach, we present a new information-theoretic view of its functioning, and propose an improvement in both theory and practice.

Since 1990 [2], these distance-dependent statistical potentials—also called “potentials of mean force” (PMF) by analogy with the potentials used in the physics of liquids [3], [4]—have been continuously applied to model quality assessment, as well as to various problems in structural biology, mainly ab initio protein folding [5], [6], [7], [8], [9], [10], [11], molecular docking [12], [13], and fold recognition [14], [15], [16]. In addition to interatomic distances, other structural features of proteins have been used, such as dihedral angle values, or solvent accessibility. Then, with the emergence of machine learning approaches, such scores resulting from the statistics of various structural descriptors have been combined into composite scoring functions [17], [18], [19], [20], [21]. Most recently, statistical potentials have drawn attention by their use in a deep learning-based approach for predicting protein structures [22].

The relatively good correlation of these scores with the free energy variation of protein folding, as well as the presence of a logarithm in the formula, have lead authors to describe this approach as resulting from the inverse Boltzmann law. However, this physical explanation has been criticized and demonstrated as being invalid on several points [23], [24], notably the fact that the atomic system of a polypeptide chain is not fairly comparable to that of a liquid. Moreover, computing both *f_i,j_*^OBS^(*r*) and *f_i,j_*^REF^(*r*) on native conformations does not allow interpreting the score as a free energy variation between the unfolded and the folded states. Another point concerns the *kT* factor, which is (most often) not taken into account and replaced by an arbitrary value, thus further invalidating the physical interpretation. In 1997, Baker and co-workers qualitatively showed that statistical potentials should actually be seen as an application of Bayes’ theorem to the conditional probabilities of pairwise distances [7]. In this view, the *f_i,j_*^OBS^(*r*)/*f_i,j_*^REF^(*r*) factor in Eq. (1) is equivalent to the ratio of the posterior to prior probabilities *p*(*r*|*i,j*)/*p*(*r*)—where *p*(*r*|*i,j*) and *p*(*r*) are the probabilities of observing two atoms at a distance *r*, with and without the knowledge of the atom types (*i* and *j*), respectively—thus quantifying the Bayesian updating. Hamelryck and co-workers later proposed a quantitative explanation [25], [26], [27], according to which statistical PMFs approximate Jeffrey's conditioning (or probability kinematics) [28], [29], an alternative updating rule. A consequence of this is the non-necessity for data in the training set to follow a Boltzmann distribution. Despite the pertinence of this probabilistic framework, the misleading justification based on physics is still recurrent in the literature (e.g. [30], [31], [32], [33], [34]), presumably because it does not interfere with the practical success of statistical potentials.

Here, we propose a new formalism that is conceptually advantageous over the popular thirty-year-old statistical potentials, as it is disconnected from any physical interpretation, while being more relevant to probabilistic reasoning. As a proof of concept, we have built two scoring functions, respectively based on the new and the PMF equations, and compared their performance at ranking predicted structures of proteins by their quality. Using the reference dataset 3DRobot (n = 60,200 structures) [35], we show that the scoring function built with our new formalism is more accurate than statistical PMFs, based on three types of performance evaluation. Finally, in our theoretical development, we also propose an explanation of what this new score measures regarding information—defined here as the quantitative property that is incorporated into the statistical model to update the prior probability.

## 2 Methods

### 2.1 Theory

There are two critical elements in Eq. (1), the first being how the reference state *f_i,j_*^REF^(*r*) is defined. The most straightforward way to do so is to calculate *f_i,j_*^REF^(*r*) as the weighted arithmetic mean of all *f_i,j_*^OBS^(*r*) [36]. It is actually the same calculation as for *f_i,j_*^OBS^(*r*), except that the atom types are indistinct. Formally, the reference state could thus be written as *f_x,x_*^OBS^(*r*), where *x* is an atom of any type. Throughout the years, various improvements have been brought to this approach (see [37] for a review and comparative test), for example by taking into account the radius of gyration of each native structures, as the size of the proteins included in the training dataset is an obvious bias for the resulting interatomic distance distributions. The other critical part in Eq. (1) is the logarithm, as it is a source of confusion between statistical potentials and Boltzmannian statistical mechanics. Boltzmann's entropy formula can be derived from classical mechanics. The logarithm thus appears when applying the second law of thermodynamics to the Hamiltonian of a model system made of a single particle moving in a U-shaped potential [38]. In statistical potentials, the logarithm was presumably introduced for computational convenience, as it maps multiplication into addition, and for facilitating interpretation of the results: e.g. log(*f_i,j_*^OBS^(*r*)/*f_i,j_*^REF^(*r*)) takes the values +1 and −1, for frequency ratios 10/1 and 1/10, respectively. However, to the best of our knowledge, it has never been raised—in the context of structural biology—that Eq. (1) is also equivalent to a calculation of a relative difference between *f_i,j_*^OBS^(*r*) and *f_i,j_*^REF^(*r*). Indeed, the statistical PMF formalism in Eq. (1) can alternatively be written (with *kT* = 1) as:

(2) $$-\text{ln}\left[\frac{f_{i,j}^{\mathrm{OBS}}\left(r\right)}{f_{i,j}^{\mathrm{REF}}\left(r\right)}\right]=-\frac{f_{i,j}^{\mathrm{OBS}}\left(r\right)-f_{i,j}^{\mathrm{REF}}\left(r\right)}{L\left[f_{i,j}^{\mathrm{OBS}}\left(r\right),f_{i,j}^{\mathrm{REF}}\left(r\right)\right]}$$

where *L*[*f_i,j_*^OBS^(*r*), *f_i,j_*^REF^(*r*)] is the logarithmic mean of *f_i,j_*^OBS^(*r*) and *f_i,j_*^REF^(*r*). Given that *f_i,j_*^REF^(*r*) is computed based on the average of all *f_i,j_*^OBS^(*r*), calculating any type of mean between the two values *f_i,j_*^OBS^(*r*) and *f_i,j_*^REF^(*r*) is irrelevant. Instead, the frequency difference *f_i,j_*^OBS^(*r*) − *f_i,j_*^REF^(*r*) should simply be divided by *f_i,j_*^REF^(*r*). The latter would then properly play its part as a reference. Therefore, we propose here to change the PMF formalism, and compute the score of a whole protein structure as:

(3) $$\mathrm{score}=-\sum_{i,j}\frac{f_{i,j}^{\mathrm{OBS}}\left(r\right)-f_{i,j}^{\mathrm{REF}}\left(r\right)}{f_{i,j}^{\mathrm{REF}}\left(r\right)}$$

In addition of being more statistically sound, this new formalism avoids any confusion with the Boltzmann distribution law, as it does no longer contain any logarithm. Another non-negligible advantage is that it does not require any “pseudo-count” calculation procedure. The latter is otherwise necessary, as the logarithm function is undefined for zero. Beyond its theoretical advantages, the practical validity of this new formalism is demonstrated in the present article through an extensive benchmarking of model quality assessment.

Since the new formalism is disconnected from physics, the score produced can no longer be viewed as an approximation of the free energy, and one may wonder what property is measured here. In what follows, we propose an explanation of how our scoring function works. In a distance distribution obtained from native conformations, in which all atom types are indistinct, observing two atoms (belonging to residues separated by at least three positions) at a distance of 2 Å can be thought surprising. However, this observation becomes less surprising, when considering only the subdistribution of the cysteine atoms, as these residues can form disulfide bonds. This decrease in the surprise is measured in both Eq. (2) and Eq. (3) by the frequency difference Δ*f*(*r*) = *f_i,j_*^OBS^(*r*) − *f_i,j_*^REF^(*r*) = *f_i,j_*^OBS^(*r*) − *f_x,x_*^OBS^(*r*). This change in the observed probabilities actually quantifies the information gain provided by the knowledge of the residue type. The more the surprise decreases, the more negative Δ*f*(*r*) is, and the more native-like is the observed interaction. Conversely, an increase in how surprising the observation is (Δ*f*(*r*) > 0) after knowing the residue type indicates a non-native interaction. To evaluate an entire protein model, all the Δ*f*(*r*) for all atom pairs in the structure have to be added. However, summing all the Δ*f*(*r*) requires distinguishing, for example, a 0.2–0.4 difference from a 0.7–0.9 one. This is achieved through a relative difference calculation, *i.e.* through dividing *f_i,j_*^OBS^(*r*) − *f_i,j_*^REF^(*r*) by a reference. In the PMF formalism as written in Eq. (2), this reference is the unnecessary logarithmic mean between *f_i,j_*^OBS^(*r*) and *f_i,j_*^REF^(*r*). In Eq. (3), we have simply replaced this logarithmic mean and used, instead, *f_i,j_*^REF^(*r*) as a reference. We named the calculated score “total information gain” (TIG), which is expressed as a dimensionless quantity.

Here, we call attention to the fact that, independently of the probabilistic framework, the property quantified by Sippl’s PMFs should also be interpreted as an information gain (rather than a pseudo-energy), but only when restricting the definition of information to the Shannon “surprisal”. Indeed, Eq. (1) can be alternatively written (with *kT* = 1) as:

(4) $$-\text{ln}\left(f_{i,j}^{\mathrm{OBS}}\left(r\right)\right)-\left[-\text{ln}\left(f_{i,j}^{\mathrm{REF}}\left(r\right)\right)\right]=I_{i,j}^{\mathrm{OBS}}\left(r\right)-I_{i,j}^{\mathrm{REF}}\left(r\right)=\Delta I_{i,j}\left(r\right)$$

where *I_i,j_*^OBS^(*r*) and *I_i,j_*^REF^(*r*) are the surprisals of observing two atoms *i* and *j* at a distance *r*, for the observed and reference distributions, respectively, and Δ*I_i,j_*(*r*) is the corresponding information gain. Thus, a total information gain is calculated by summing every Δ*I_i,j_*(*r*) for every combination of atoms *i* and *j* found in the evaluated structural model. Also of note is the fact that the variation of Shannon entropy (*i.e.* the average amount of information) is simply obtained by dividing this total information gain by the total number of interatomic distances in the evaluated protein structure. However, this aspect will not be developed further in the present article.

Finally, to further demonstrate the irrelevance of the logarithmic mean in Eq. (2), we have built two “mock” scoring functions, in which this mean is replaced by either the arithmetic mean of *f_i,j_*^OBS^(*r*) and *f_i,j_*^REF^(*r*), or only the highest of the two frequencies. We refer to these scores as “MCK1” and “MCK2”, respectively. Formally, they are expressed as:

(5) $$\mathrm{mock}\;score\text{1}=-\sum_{i,j}\frac{f_{i,j}^{\mathrm{OBS}}\left(r\right)-f_{i,j}^{\mathrm{REF}}\left(r\right)}{\left[f_{i,j}^{\mathrm{OBS}}\left(r\right)+f_{i,j}^{\mathrm{REF}}\left(r\right)\right]/\text{2}}$$

(6) $$\mathrm{mock}\;score\text{2}=-\sum_{i,j}\frac{f_{i,j}^{\mathrm{OBS}}\left(r\right)-f_{i,j}^{\mathrm{REF}}\left(r\right)}{max\left[f_{i,j}^{\mathrm{OBS}}\left(r\right),f_{i,j}^{\mathrm{REF}}\left(r\right)\right]}$$

and their accuracies have been measured in the benchmarking procedure described below.

### 2.2 Implementation and training procedure

To test the formalism of Eq. (3), we have modified the C++ open source code of MyPMFs [39], a computational tool from our previous work, which allows users to generate PMFs from any dataset of protein structures. The source code used for the present article is freely available for download, from the RPBS repository, at https://gitlab.rpbs.univ-paris-diderot.fr/src/ig-score.

To build our scoring function, we have defined a set of native protein structures as follows: (i) from the PISCES website (http://dunbrack.fccc.edu/PISCES.php) we have downloaded a precompiled list of 3768 PDB chains of resolution ≤1.6 Å (X-ray structures only), R-factor ≤ 0.25, and sequence identity ≤20%; (ii) we only kept the 1973 protein chains of lengths ranging from 80 to 250 residues; (iii) to ensure independence from the benchmark dataset, we have eliminated the 56 protein chains that share >20% sequence identity with any of the 200 proteins from the 3DRobot dataset [35]. This last step has been carried out using the standalone version of PISCES [40]. The resulting list of 1917 protein chains is available in the supplementary materials.

To compare the formalisms of Eqs. (1) and (3), we have trained two scoring functions, to which we will refer as “PMF” and “TIG”, respectively. For both, the reference state *f_i,j_*^REF^(*r*) has been calculated as the weighted arithmetic mean of all *f_i,j_*^OBS^(*r*) [36], using all-atom representation of the native structures. The interatomic distance distributions have been computed for distance bins of 0.5 Å, and a distance cutoff of 15.0 Å. The distances between atoms belonging to residues *i* and *i* + 1, *i* + 2, or *i* + 3 have not been taken into account [41]. The frequencies have been obtained by using kernel density estimations, as implemented in the R standard library (version 3.2.3). The bandwidths of the Gaussian kernels have been selected with the Scott’s rule-of-thumb [42]. The same procedure has been followed for the MCK1 and MCK2 scores.

### 2.3 Benchmarking procedure

Each scoring function has been assessed based on its ability to rank structural models by quality as measured by their TM-score [43] to the native structure, which takes values between 0 and 1 (the higher the TM-score, the higher the model quality). As a benchmark, we used the 60,200 structures from the 3DRobot dataset [35], which represents 200 non-homologous proteins (48 α-, 40 β-, and 112 α/β-single-domain structures), each having 300 decoys and 1 native conformation. Additionally, we used predicted protein structures from the CASP13 experiment (2018). We selected models corresponding to targets in both the template-based modeling and free modeling categories, taking every model produced by every group. This represents a total of 52,296 models from 133 targets.

Each scoring function was evaluated through a pairwise ranking of the decoys for each of the 200 proteins from 3DRobot, or each of the 133 CASP13 targets. This allowed to calculate the accuracy of each method as the proportion (in %) of correct pairwise rankings. As the difficulty of ranking models may vary depending on their qualities, four subsets of the 3DRobot and CASP13 datasets have been defined, based on the TM-score to the native: “near-native”, “good”, “medium”, and “poor” quality models are defined by three thresholds at 0.8, 0.6, 0.4, respectively. Since comparing two very similar models is pointless, another threshold for the minimal TM-score difference between the compared models has been defined at 0.1. The other performance criterion used in this study is the average ranking, as predicted by the scoring function, for the aforementioned “near-native” and “good” categories of models (the higher the rank, the better), as well as for the “poor” ones (the lower the rank, the better). The statistical significance of the observed differences between accuracies was determined by comparing the distributions of correct and wrong rankings, using the Wilcoxon signed-rank test, with an α error of 0.05. The exact same procedure has been carried out using the global distance test total score (GDT_TS) [44] instead of the TM-score. Since these two measures are calculated on the Cα of the protein structures, the scoring with the statistical potentials was restricted to this atom type.

To compare both the PMF and TIG scores with an external reference from the literature, we have included the DOPE [45] and GOAP scores [46] into the benchmark. The former is the most cited of all model quality assessment programs, while the latter is a more recent and high-performing statistical potential. Similarly to the scoring functions that we have built here, the only structural features that are quantified by DOPE are the interatomic distances, using the same distance bins and cutoff (0.5 Å and 15.0 Å, respectively; see above). GOAP is both distance- and angle-dependent: for each heavy atom in interacting pairs, it uses the relative orientation of the corresponding planes. For the computing *f_i,j_*^REF^(*r*), DOPE and GOAP take into account either the radius of gyration or the molecular volume of each protein structure from the training set, which eliminates the bias on the interatomic distances—the distance distributions may vary a lot, depending on the sizes of the proteins included in the dataset. Thus, the difference between DOPE/GOAP and PMF/TIG lies in (i) the training datasets, (ii) the calculation of the reference state and (iii), only in the case of GOAP, the dependence on orientation.

## 3 Results and discussion

### 3.1 Performance: pairwise model ranking

The TIG scoring function is supposed to be more accurate than Sippl’s statistical potentials (Eq. (1)), as it is built on the new approach (Eq. (3)). To demonstrate its practical superiority, we have compared its performance with those of our PMF score, through two different tests. The first one evaluates the ability of the method to rank pairs of models taken from the 3DRobot dataset. The results of this benchmarking procedure are presented in Table 1. They include the accuracies of the DOPE, MCK1, and MCK2 scores (for GOAP, see the next paragraph). Overall, it appears that TIG is the best of these five methods, whereas PMF is the worst. To our surprise, the two mock scores systematically outperform PMF, although they were designed only to prove the irrelevance of combining *f_i,j_*^OBS^(*r*) and *f_i,j_*^REF^(*r*) as a statistical reference. On the whole, the results obtained with the mock scores are also significantly better than those produced by DOPE. This indicates that the logarithmic mean could be advantageously replaced by other types of means, such as the arithmetic mean used in MCK1.

**Table 1:** Accuracy in ranking pairs of decoy structures from 3DRobot. A 50.0% value would correspond to a random ranking. The “near-native”, “good”, “medium”, and “poor” model qualities correspond to score (TM-score or GDT_TS) intervals [1.0, 0.8[, [0.8, 0.6[, [0.6, 0.4[, and [0.4, 0.0], respectively.

		Accuracy (%)					
Model quality	Score	PMF	MCK1	MCK2	DOPE	TIG	GOAP
Near-native	TM-score	67.8	69.5	70.3	67.0	71.6	91.5
Good		70.6	73.3	74.2	71.4	75.1	86.8
Medium		71.2	72.9	73.6	75.6	75.2	80.8
Poor		68.4	69.2	70.1	71.4	70.6	76.2

Near-native	GDT_TS	63.5	64.8	65.7	61.8	67.0	88.0
Good		68.3	69.7	70.6	67.9	70.8	85.3
Medium		73.5	75.3	75.8	76.5	77.0	86.5
Poor		66.8	68.3	68.8	71.1	70.2	76.0

This article describes how Sippl’s formalism can be comprehended and improved, in light of probability and information theories. The simple TIG and mock scores have been designed for that purpose. However, to give the reader an idea of the performance that a more complex scoring function can achieve, our benchmark includes a sixth method, GOAP, which ranks among the best statistical potentials [46]. The results obtained with GOAP are dramatically better than those produced with any of the other methods. In particular, GOAP outperforms TIG by ~20% on the near-native decoys. On the “Good” category, this difference is still >10%, when taking either the TM-score or the GDT_TS as a reference. On the two other categories of model quality, GOAP is also the most accurate method. This significant superiority is consistent with what has been previously observed on other datasets of decoys [46], where GOAP outperformed the OPUS-PSP potential [47] by ~15%. The latter was itself reported as more accurate than statistical potentials of lower complexity (*i.e.* which use less information), such as DFIRE [48], RWplus [49], and dDFIRE [50], [51]. Thus, the results obtained with GOAP were expected and can be explained by the greater amount of stereochemical information it uses: the orientation and, in a lesser extent, the volume of the protein molecules. Here, it should be highlighted that the only unbiased comparison—to provide insights into the improvement brought about by the new formalism—is the one between PMF and TIG, as these scoring functions are equally complex. It is also the case for the two mock scores, which have been specially designed for the sake of fair comparison.

**Fig. 1:**
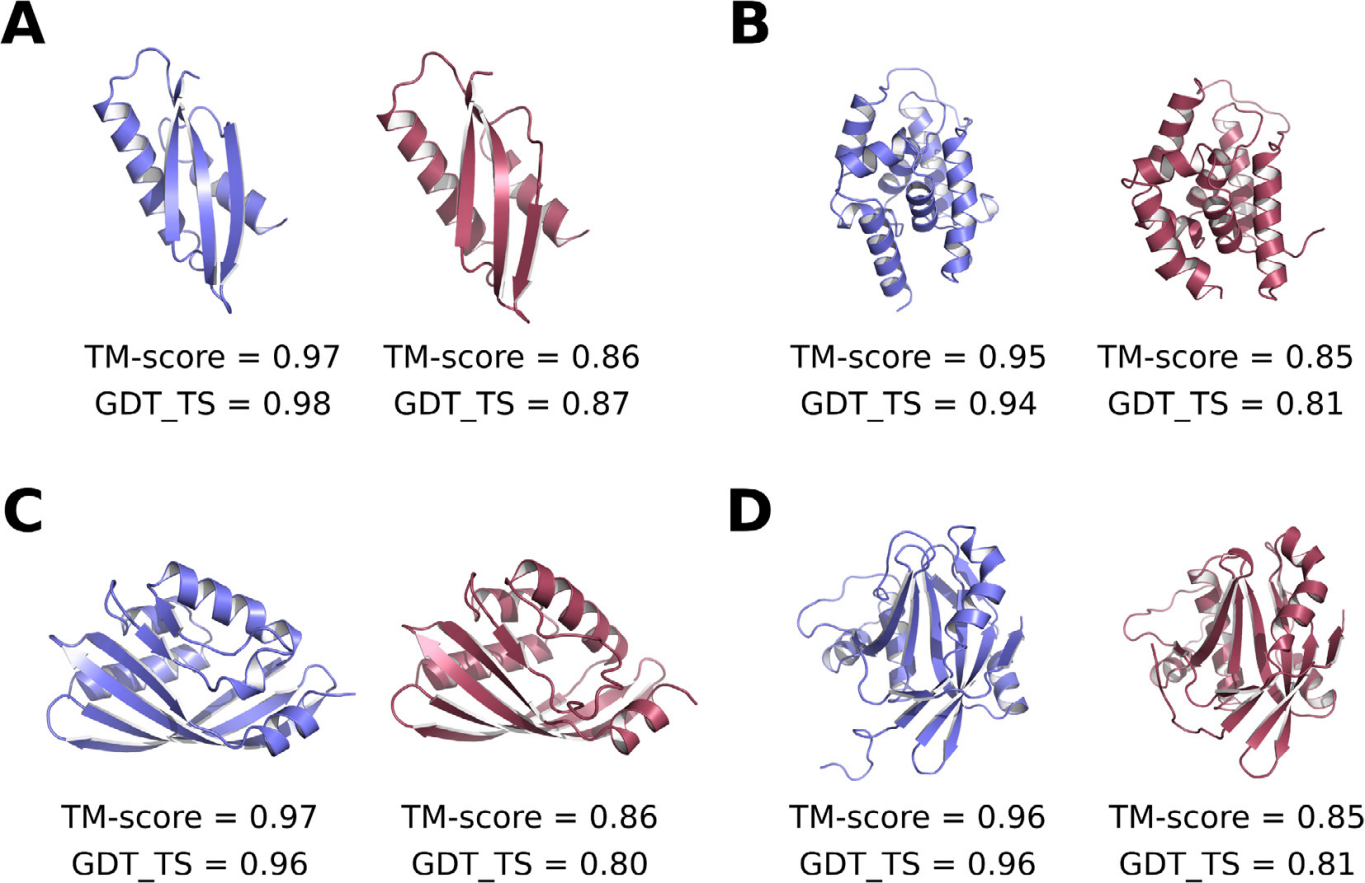
Examples of protein models correctly and incorrectly ranked with the information-gain based approach, TIG. For each example, the better and worse models are represented in blue and red, respectively. (A) Predicted structures of the CASP13 target T1006 (magnetosome protein MamM) correctly ranked by TIG, but incorrectly ranked by the PMF, mock, and DOPE scoring functions. (B) Decoy structures of the ATP-binding subunit ClpC1 of the Clp protease (PDB code 3wdeA) from the 3DRobot dataset, which are correctly ranked by all methods except TIG. (C) Predicted structures of the target T0971 (terfestatin biosynthesis enzyme TerC), for which only TIG fails. (D) Decoy structures of the DUB domain of the human zinc metalloprotease AMSH-LP (PDB code 2znrA), for which only TIG succeeds. (For interpretation of the references to colour in this figure legend, the reader is referred to the web version of this article.)

The ranking of the methods is similar to the CASP13 benchmark, although every accuracy is higher (Table S1). On this dataset, PMF is systematically outperformed by the two mock scores, which are themselves outperformed by TIG. The latter is not significantly better than DOPE on this dataset, but GOAP is still far more accurate than the other five methods. Compared to 3DRobot, the differences between the methods are aggravated for the poor quality models, whereas they are non-significant for the near-native ones. Moreover, the quality of the models appears to have a different influence over the accuracy: (i) the most difficult models to rank are those of near-native and poor quality in 3DRobot versus those of medium quality in CASP13; (ii) the easiest to rank are those of good and medium quality in 3DRobot versus those of near-native quality in CASP13. Only the performances of GOAP are consistent between the two datasets: the higher the model quality, the higher the accuracy. All these discrepancies presumably arise from the different origins of the two datasets. The 3DRobot set has been specifically designed for benchmarking purposes: for each protein, the native structure has been uniformly altered to generate exactly 300 decoys. Models from CASP13 are produced by different competing research groups, so that there is no control over their quality, nor over their number. Nevertheless, both series of results lead to the same conclusion regarding the improvement brought about the TIG formalism over the statistical PMFs. Finally, although not statistically balanced, the CASP13 dataset provides actual predictions of protein structures, unlike the decoys of 3DRobots, *i.e.* native structures of altered quality. Thus, cases of success and failure of TIG, selected from both CASP13 and 3DRobot, are presented in Fig. 1.

### 3.2 Performance: average predicted rank

To confirm the above benchmarking results, a second test has been performed. It consists in observing how the models are ranked by a scoring function, depending on their actual quality as measured by either the TM-score or the GDT_TS. The results of this second benchmarking procedure are presented in Table 2. For the “near-native” and “good” models, the lower the value presented in Table 2 (*i.e.* the higher the rank), the better; conversely, for models of “poor” quality, the lower the rank, the better. Taken as a whole, these results further validate the new formalism, as TIG significantly outperforms the other methods except GOAP. Indeed, TIG is better because it (i) ranks higher the near-native models, as well as the good models (although only when defined with the GDT_TS) and (ii) ranks lower the models of poor quality. Again, the mock scores systematically outperform PMF, and DOPE is the only score that rivals or bests TIG (at ranking good models). In agreement with the accuracies reported in Table 1, the performance of GOAP is far superior to those of the other methods, on the near-native and poor models. This is, however, not the case for the good models, which shows that the quality category influences the average predicted rank differently than the accuracy. In general, this second test is less discriminating than the pairwise ranking, since the observed differences are less significant.

**Table 2:** Ranks predicted by the TIG, DOPE, mock, and PMF scores, averaged for three categories of models. For the “near-native” and “good” models, the lower the value (the higher the rank), the better the performance. Conversely, for models of “poor” quality, the lower the rank, the better.

		Average predicted rank					
Model quality	Score	PMF	MCK1	MCK2	DOPE	TIG	GOAP
Near-native	TM-score	68.1	64.8	63.9	67.1	61.9	46.3
Good		123.4	122.3	122.2	120.5	121.9	123.6
Near-native	GDT_TS	63.1	59.5	58.4	61.5	55.9	38.4
Good		102.5	100.6	100.2	101.0	100.0	98.1
Poor	TM-score	222.9	226.2	227.0	229.1	229.3	237.3
Poor	GDT_TS	222.0	224.9	225.5	226.3	227.3	234.8

As for the first test, the results contain some discrepancies between the TM-score- and GTD_TS-based categories. This shows the difficulty of defining thresholds to categorize model quality. As both the TM-score and GDT_TS take values between 0 and 1, we used the same thresholds for these two scores. The >0.8 limit for the near-native models was defined based on a major and recent study, which defined conformations with a TM-score >0.7 as “high-accuracy” predicted structures [22]. We defined the <0.4 limit for the models of poor quality, based on the previously studied significance of a TM-score of 0.5 [52]. However, our categorization of the model quality seems appropriate, given that the tested methods have more difficulty ranking the near-native and poor models (for different reasons), than the ones of good or medium quality.

### 3.3 Performance: correlation between predicted and true quality measures

**Fig. 2:**
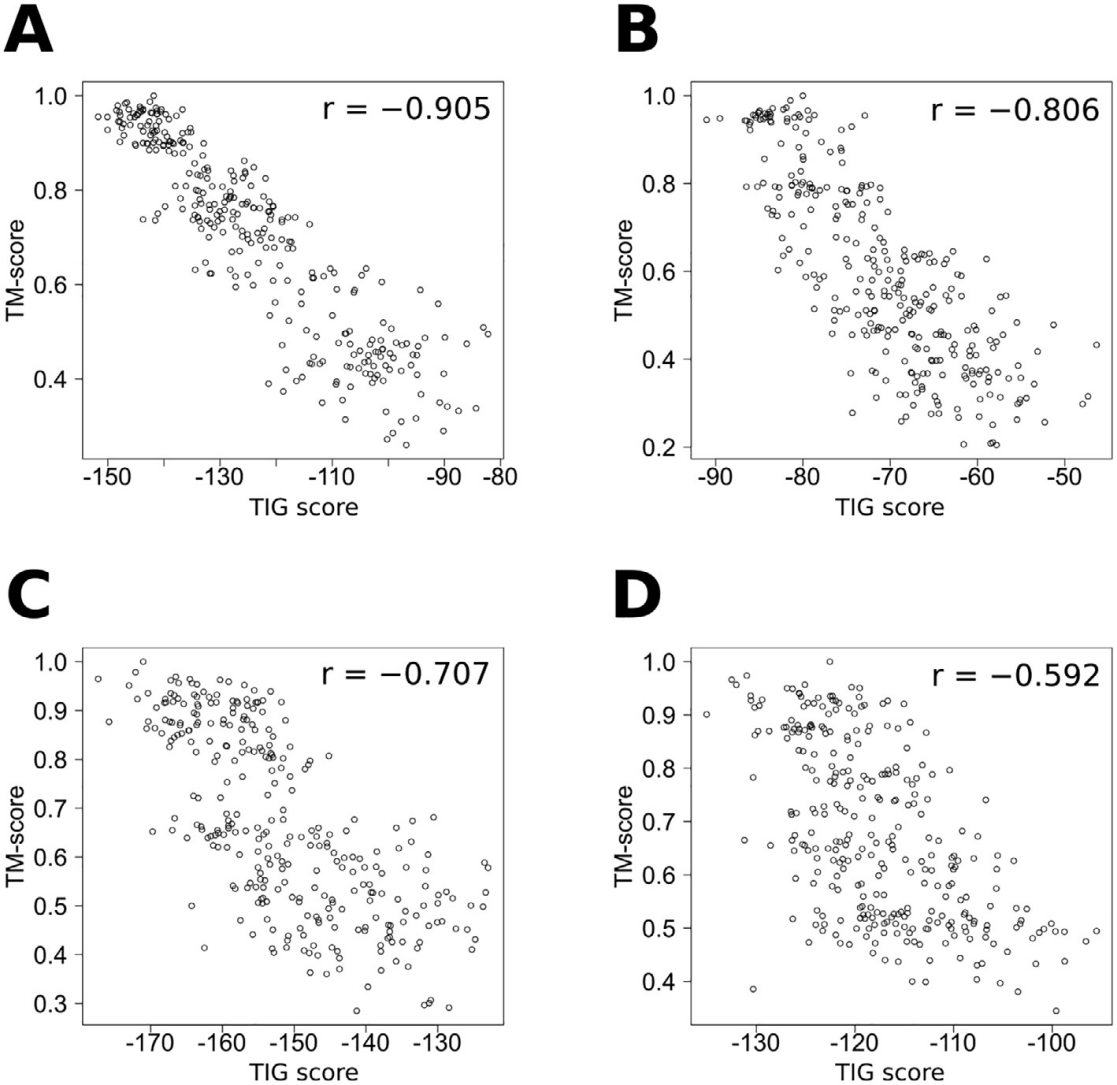
Predicted quality (TIG score) of decoy structures from 3DRobot plotted against their true quality (TM-score). The Pearson correlation coefficient *r* is given for each example. (A) Conserved domain of nonstructural protein 3 (nsP3) from SARS coronavirus (PDB code 2acfA; 182 residues). (B) Dihydroneopterin aldolase from *Escherichia coli* (PDB code 2o90A; 122 residues). (C) Catalytic domain of the DNA glycosylase MutY (PDB code 1munA; 225 residues). (D) Protoglobin from *Methanosarcina acetivorans* (PDB code 3qzxA; 195 residues).

To further validate the information gain-based approach, the correlations between the scores produced by PMF or TIG and the corresponding TM-scores have been investigated, for the decoys of 3DRobot. Averaged on the 200 proteins (×300 decoys), the Pearson correlation coefficients are −0.719 and −0.782, for PMF and TIG, respectively. This makes TIG equal to dDFIRE, by referring to performances previously reported in the literature [53]. Like GOAP, the dDFIRE statistical potential is both orientation- and distance-dependent, and uses protein molecular volume in the calculation of the reference state. However, unlike GOAP, it is based on a coarse grained representation of the protein structures. It should be noted here that dDFIRE is the lowest-performing program among those tested in [53], where SVMQA is the best, followed by OPUS-PSP, GOAP, and RWplus. Nevertheless, the outcome of this comparison is that TIG can match a more complex method, such as dDFIRE. These results also confirm the improvement brought about the TIG formalism over Sippl’s PMFs. Finally, as the 200 proteins from 3DRobot have been selected for their diversity, the difficulty to assess the decoys may vary from one protein to another. This is illustrated by Fig. 2, in which values of the TM-score are plotted against those of the TIG score, for proteins that show various levels of correlation. In these examples, the dispersion of the predicted quality goes higher, as the true quality goes lower. This is consistent with the intuition that the more altered is a decoy structure, the more uncertain is the prediction of its quality.

### 3.4 Qualitative analysis: score profiles

**Fig. 3:**
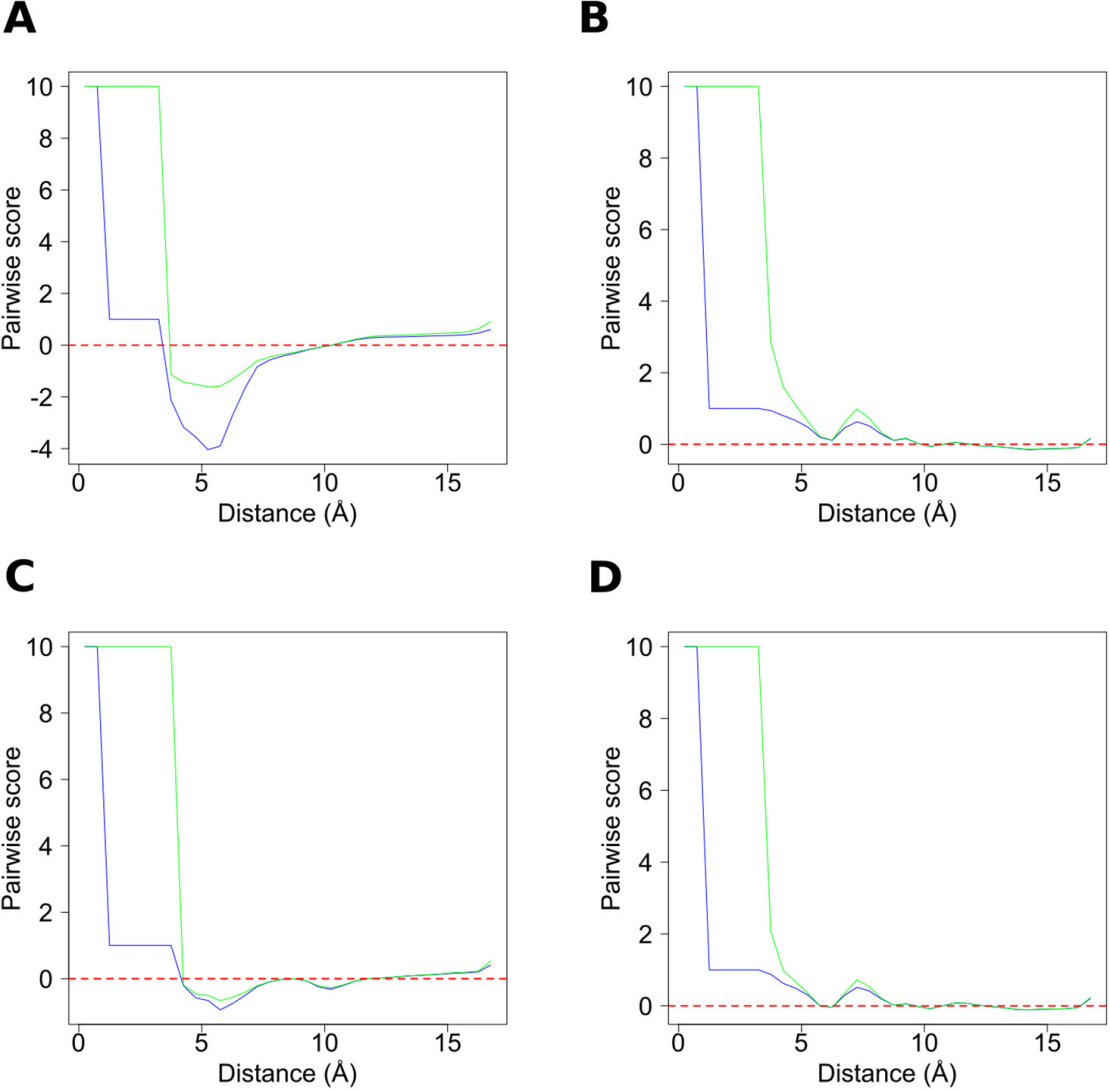
Score profiles from the TIG (blue) and PMF (green) methods. The interacting atoms are the Cα of the (A) Cys-Cys, (B) Asp-Glu, (C) Val-Val, and (D) Lys-Arg residue pairs. (For interpretation of the references to colour in this figure legend, the reader is referred to the web version of this article.)

As the new formalism has been designed to be independent of any physics-based interpretation, it is interesting to analyze the score profiles of our TIG function, given that we will not attempt to draw any analogy with physical interatomic potentials (e.g. the Lennard-Jones or Morse potentials). Compared with the PMF profiles, a first observation is that most profiles are actually very similar. Therefore, Fig. 3 presents the TIG and PMF profiles for the four pairs of residues that differ the most, namely the Cys-Cys, Asp-Glu, Val-Val, and Lys-Arg residue pairs. Strikingly, one can observe that the attractive part of the profile (*i.e.* the negative score well) is always stronger for TIG than for PMF. This is simply due to the fact that, for *x* and *y* ∈ ]0, 1], the −ln(*x*/*y*) function from Eq. (1) takes values that are always greater than those of −(*x* − *y*)/*y* from Eq. (3). Therefore, the scores computed are systematically lower with TIG than with PMF. It is important to note that this difference is not related to the better performance of the new formalism, as our benchmark was only aimed at testing the ability of the scoring functions to rank models, rather than to assess their absolute quality. In other words, it is not valid to compare the two scores computed by TIG and PMF for each structure, and conclude that TIG always evaluates the structure as more native-like.

Another remarkable feature of the score profiles concerns their repulsive part, on the left side of each plot. Both TIG and PMF functions are undefined for *f_i,j_*^REF^(*r*) = 0, which requires arbitrarily setting a default value of the score (here equal to +10). However, due to the logarithm in the formula, only PMF is undefined for *f_i,j_*^OBS^(*r*) = 0, which also requires a default value (again set to +10). For *f_i,j_*^OBS^(*r*) = 0, the TIG scoring function is defined and takes the value +1, which makes its repulsive part composed of two plateaus, at +1 and +10. The +1 plateau corresponds to an interatomic distance that has never been observed for the particular atom pair, but otherwise exists in the training set of native conformations. The +10 plateau, however, corresponds to an interatomic distance that has never been observed within the experimental structures, whatever the type of atoms. Although it seems useful to distinguish these two cases, with a higher penalty for the second one, this repulsive part with either one or two plateaus presumably does not affect the results of our benchmarking procedure. Indeed, only protein models of very poor quality would contain such abnormal interatomic distances. Nevertheless, the importance of this parameter—which can be applied to both TIG and PMF formalisms—remains to be investigated. Interestingly, the right sides of the plots indicate that the information gain is limited for two residues spaced by >10 Å, as the score fluctuates around zero. This suggests that similar performances could be achieved at a lower computational cost, by restricting calculations to shorter interatomic distances (see section below). Finally, from a qualitative point of view, these profiles produced by TIG do not seem to show any unexpected features. For the Asp-Glu and Lys-Arg residue pairs (Fig. 1B and D, respectively), the positive peak at ~7 Å is consistent with their presumably repulsive interaction. Similarly, in the Val-Val (Fig. 1C) profile, the negative well at ~6 Å can be attributed to the attractive interaction between two hydrophobic residues, and the very negative profile of the Cys-Cys (Fig. 1A) pair reflects the possibility of forming disulfide bonds.

## 4 Conclusions and perspectives

The new formalism presented here was developed to be more statistically relevant than Sippl’s PMFs. Thus, the better performances observed on the benchmark were actually expected. Through the inclusion of the two mock scoring functions, this study was also aimed at shedding light on how the statistical PMFs actually work by summing relative frequency differences, which correspond to information gains. It should be noted that we used here a general definition of information that quantifies the Bayesian updating and is, therefore, different than the particular Shannon surprisal (also called “self-information”). Importantly, the conceptual improvement brought here is only valid when *f_i,j_*^OBS^(*r*) is computed from a subdistribution of *f_i,j_*^REF^(*r*). When the prior and posterior distributions are of equal complexity, the logarithmic mean of *f_i,j_*^OBS^(*r*) and *f_i,j_*^REF^(*r*) holds relevant. However, the advantage of dividing by a logarithmic mean in Eq. (2), rather than by a generalized mean (the special cases of which being the arithmetic, geometric, and harmonic means) still has to be demonstrated.

In their original form, as devised by Sippl thirty years ago, the statistical potentials used only the distance between each pair of atoms to represent protein structures. Through the lens of probability theory, Bakers’s and Hamelryck’s research groups later showed how any other descriptors can be successfully used: typically, solvent accessibility or torsion angles. More exotic structural properties have also been exploited, e.g. lipid bilayer depth to build a potential aimed at evaluating structural models of transmembrane proteins [54]. Nevertheless, in the particular case of scoring functions that are only based on interatomic distances (like TIG), the performances might find their roots not only in Bayes’ theorem, but also in the representation of the problem. Reducing a protein 3D structure to a set of pairwise distances allows the use of graph theory. Protein conformations can thus be modeled as amino acid (weighted or unweighted) graphs and are referred to as “protein contact networks” (PCNs; see [55] for a review). Similarly to the TIG formalism, methods based on PCNs are not related to physics and, yet, are able to rank decoys [56]. Authors have later combined such graph-theoretic approach with support vector machine in order to accurately assess the quality of structural models [57]. As a consequence of these results, generalizing the TIG concept and confirming its relevance regarding statistics would require to rule out the PCN representation as a source of performance. This would mean evaluating the accuracy of TIG scoring functions that would be built on other structural features than distances. The resulting scoring functions could be used as knowledge-based terms, combined with physics-based terms, into a composite energy function, such as that developed for the Rosetta modeling software [58]. The weight of all terms would be optimized to fit experimental structural and thermodynamic data. Alternatively, the elementary scores could be included in non-linear statistical models, thanks to machine learning and deep learning techniques, as it can yield highly accurate quality assessment programs [53], [59], [60].

The development of a distance-dependent scoring function relies on several parameters, such as the distance bin width, the minimum and maximum distance thresholds, and the minimum number of sequence positions separating the residues of the two interacting atoms. Behind the setting of these values lies the question of how to treat long-range interactions and local contacts. Here, we considered distances ranging from 0 to 15 Å in order to be comparable with DOPE, but authors use a 4–8 Å range, following the aforementioned PCNs approach [55]. Attempts to determine optimal values for these parameters have been made [61]. However, the training dataset and, more importantly, the benchmark then used were too small to draw any permanent conclusion. Such a study could be redone with the computational tool used for developing the TIG score, as it allows to create a custom scoring function, while setting the different thresholds with user-selected values. Interestingly, the results obtained with GOAP show that a method based only on interatomic distances, orientation, and molecular volume can achieve high accuracy—especially for near-native decoys. This would indicate that a limited number of parameters are sufficient to model the process of protein folding and stability. Such knowledge-based scoring functions, as estimators of protein free energy, could thus be considered as “sloppy models”, *i.e.* models whose behavior depends on a relatively small number of combinations of parameters. Although this theoretical framework has gained popularity in recent years for explaining phenomena in physics and biology (see [62]), it remains unused as a means to study protein structures.

We proved our concept for the ranking of predicted structures according to their quality. However, there are several other applications that could be explored—like protein–protein docking, for example. An interesting use of statistical potentials consists in training them on a particular type of native protein conformations, in order to gain insight into the rules that govern the relative positioning of residues within these protein structures. For example, this has recently been done for transmembrane protein structures [63]. As we provide here an open-source standalone version of our program, we hope that it will find usefulness in studying pairwise interactions within user-selected protein structures. Finally, this study focused on statistical pairwise potentials. Similarly to their physics-based counterparts, these two-body potentials are inherently limited in their representation of the intra-protein interactions. Further investigations should therefore be carried on many-body potentials.

## CRediT authorship contribution statement

**Guillaume Postic:** Conceptualization, Formal analysis, Methodology, Software, Supervision, Validation, Writing - original draft, Writing - review & editing. **Nathalie Janel:** Funding acquisition, Writing - review & editing. **Pierre Tufféry:** Formal analysis, Funding acquisition, Validation, Writing - review & editing. **Gautier Moroy:** Formal analysis, Validation, Writing - original draft, Writing - review & editing.

## Declaration of Competing Interest

The authors declare that they have no known competing financial interests or personal relationships that could have appeared to influence the work reported in this paper.
