# Supplementary material for: An information gain-based approach for evaluating protein structure models
Source: Comput Struct Biotechnol J. 2020 Aug 18;18:2228–36. doi: 10.1016/j.csbj.2020.08.013 (PMC7431362; doi:10.1016/j.csbj.2020.08.013)
Supplement: Supplementary Data 1 [file mmc1.doc]

**SUPPLEMENTARY MATERIAL**

**An information gain-based approach for evaluating protein structure models**

Guillaume Postic^1,2,3,4^*, Nathalie Janel^2^, Pierre Tufféry^1,4^, Gautier Moroy^1^

^1^Université de Paris, BFA, UMR 8251, CNRS, ERL U1133, Inserm, F-75013 Paris, France

^2^Université de Paris, BFA, UMR 8251, CNRS, F-75013 Paris, France

^3^Institut Français de Bioinformatique (IFB), UMS 3601-CNRS, Université Paris-Saclay, Orsay, France

^4^Ressource Parisienne en Bioinformatique Structurale (RPBS), Paris, France

*To whom correspondence should be addressed: [guillaume.postic@univ-paris-diderot.fr](mailto:guillaume.postic@univ-paris-diderot.fr)

**Table S1**. Accuracy in ranking pairs of decoy structures from CASP13. A 50.0% value would correspond to a random ranking. The “near-native”, “good”, “medium”, and “poor” model qualities correspond to score (TM-score or GDT_TS) intervals [1.0, 0.8[, [0.8, 0.6[, [0.6, 0.4[, and [0.4, 0.0], respectively.

**Training dataset**:

1A62A; 1AH7A; 1ATGA; 1B0BA; 1BGFA; 1BKRA; 1BTEA; 1BTKA; 1BYIA; 1C1KA; 1C5EA; 1C7KA; 1CCWC; 1CXQA; 1CY5A; 1DFMA; 1DG6A; 1E29A; 1E58A; 1E5KA; 1E7LA; 1EAQA; 1EB6A; 1ELKA; 1EUWA; 1EZGA; 1F1EA; 1F46A; 1FCYA; 1FM0E; 1FSGA; 1FT5A; 1FYEA; 1G2RA; 1G3PA; 1G61A; 1G6GA; 1GMUA; 1GMXA; 1GP0A; 1GPPA; 1GVPA; 1GWMA; 1GY7A; 1H2CA; 1H4XA; 1H97B; 1H99A; 1HPGA; 1HQ1A; 1HW1A; 1HXIA; 1HZTA; 1I4UA; 1I5GA; 1ID0A; 1IDPA; 1IFRA; 1IO0A; 1IQZA; 1IX9A; 1J0PA; 1J3AA; 1J3WA; 1J77A; 1J98A; 1JB3A; 1JBEA; 1JF8A; 1JHJA; 1JI7A; 1JKEA; 1JL1A; 1JNIA; 1JO0B; 1K4IA; 1K4NA; 1K7CA; 1K7JA; 1KAFA; 1KGDA; 1KMTA; 1KNMA; 1KOEA; 1KQ6A; 1KQFC; 1KT6A; 1KYFA; 1L3KA; 1L6RA; 1LMIA; 1LNIA; 1LQVA; 1LU4A; 1LWBA; 1M1FA; 1M2DA; 1M55A; 1M9ZA; 1MC2A; 1MK0A; 1MKKA; 1MN8A; 1MUNA; 1MWQB; 1MY7A; 1N08A; 1N13B; 1N62A; 1N8VA; 1NC7A; 1NG6A; 1NKIA; 1NLQA; 1NNLA; 1NNXA; 1NU0A; 1NWZA; 1NXMA; 1NYCA; 1NZ0A; 1O7IA; 1OCYA; 1OD3A; 1OI0A; 1OKIA; 1OQJA; 1OU8B; 1OW4A; 1P6OA; 1P9HA; 1PKHA; 1PMHX; 1PP0A; 1PSRA; 1PZ4A; 1Q5YA; 1Q7LB; 1QFTA; 1QGVA; 1QV1A; 1QW2A; 1R29A; 1R6JA; 1R7JA; 1RFYA; 1RG8B; 1RKIA; 1RKUA; 1ROCA; 1RTTA; 1RYLA; 1S29A; 1S2OA; 1S3CA; 1S9UA; 1SAUA; 1SENA; 1SFSA; 1SQSA; 1SX5A; 1SZ7A; 1SZHA; 1T3YA; 1T61A; 1T6UA; 1T92A; 1T9IA; 1TKEA; 1TP6A; 1TQGA; 1TT8A; 1TU9A; 1TUAA; 1U07A; 1U7IA; 1U84A; 1UCDA; 1UFYA; 1UGIA; 1UKFA; 1UNQA; 1UUYA; 1UZ3A; 1UZKA; 1V05A; 1V2XA; 1V4PA; 1V70A; 1VD6A; 1VE4A; 1VH5A; 1VHTA; 1VK1A; 1VKEA; 1VKKA; 1VL7A; 1VMGA; 1VMHA; 1VP8A; 1VQSA; 1VR7A; 1VYIA; 1VYKA; 1W0HA; 1W0NA; 1W1HA; 1W4SA; 1W53A; 1W66A; 1WHIA; 1WKQA; 1WLZA; 1WMHA; 1WN2A; 1WNAA; 1WPAA; 1WPNA; 1WPUA; 1WS8A; 1WVQA; 1WWIA; 1WZDA; 1X0TA; 1X6IB; 1X6OA; 1X6ZA; 1X8QA; 1X91A; 1XBIA; 1XD3A; 1XG0C; 1XLQA; 1XMTA; 1XPPA; 1Y43B; 1Y5HA; 1Y6XA; 1Y93A; 1Y9LA; 1YB3A; 1YD0A; 1YLXA; 1YN3A; 1YPYA; 1Z0NA; 1Z0WA; 1Z2UA; 1Z3XA; 1Z67A; 1Z6MA; 1Z6NA; 1Z72A; 1ZCEA; 1ZHVA; 1ZI8A; 1ZK5A; 1ZKEA; 1ZZKA; 2A0BA; 2A35A; 2A6ZA; 2ACFA; 2AIBA; 2ANXA; 2AP3A; 2ARCA; 2ASKA; 2AXWA; 2B0AA; 2B82A; 2BBRA; 2BCMB; 2BDRA; 2BK9A; 2BKFA; 2BKXA; 2BL8A; 2BMOB; 2BO9B; 2BRFA; 2BT9A; 2BZ1A; 2C2UA; 2C3VA; 2C60A; 2C71A; 2C8SA; 2C92A; 2CARA; 2CB8A; 2CCQA; 2CCVA; 2CDPA; 2CE2X; 2CG7A; 2CIOB; 2CIUA; 2CJTA; 2CKKA; 2COVD; 2CVEA; 2CWSA; 2CYJA; 2CZSA; 2D3DA; 2D5MA; 2D68A; 2DKOA; 2DKOB; 2DLBA; 2DTJA; 2DXAA; 2DXUA; 2DY0A; 2E3HA; 2EGVA; 2EH3A; 2EHPA; 2ENDA; 2ERFA; 2EV1A; 2EW0A; 2F01A; 2F22A; 2F23A; 2F46A; 2F5TX; 2F62A; 2F9HA; 2FB6A; 2FCJA; 2FCLA; 2FCOA; 2FCWA; 2FCWB; 2FHZA; 2FHZB; 2FJ8A; 2FKKA; 2FP1A; 2FR5A; 2FRGP; 2FSQA; 2FULA; 2FUPA; 2FWHA; 2G3RA; 2G7SA; 2GKGA; 2GKPA; 2GLZA; 2GPIA; 2GRRB; 2GS5A; 2GU9A; 2GUDA; 2GUIA; 2GXQA; 2GYQA; 2GZ4A; 2H8EA; 2HEWF; 2HIYA; 2HLYA; 2HQXA; 2HS1A; 2HUHA; 2HW2A; 2HX0A; 2HX5A; 2I3DA; 2I5UA; 2I8TA; 2IA1A; 2IA7A; 2IAYA; 2IBDA; 2IBNA; 2IC2A; 2IMFA; 2IMHA; 2IMJA; 2INWA; 2IP6A; 2IT2A; 2IU5A; 2IYVA; 2IZ6A; 2J1VA; 2J2JA; 2J43A; 2J6BA; 2J73A; 2J8KA; 2J9WA; 2JCBA; 2JDAA; 2JEKA; 2JFRA; 2JKUA; 2JLIA; 2MCMA; 2NLRA; 2NLVA; 2NMLA; 2NN5A; 2NNUA; 2NQWA; 2NR7A; 2NRRA; 2NSZA; 2NVHA; 2NWFA; 2NXVA; 2O1QA; 2O2XA; 2O7AA; 2O90A; 2O9UX; 2OB5A; 2OCTA; 2ODIA; 2ODKA; 2OFCA; 2OFKA; 2OFZA; 2OHWA; 2OIZD; 2OKFA; 2OLMA; 2OMLA; 2OMZB; 2OPCA; 2OPLA; 2OQZA; 2OV0A; 2OVJA; 2OXGY; 2OXGZ; 2OY9A; 2OYOA; 2OZHA; 2P0NA; 2P0SA; 2P14A; 2P6WA; 2P8IA; 2PA7A; 2PAGA; 2PC1A; 2PEZA; 2PFIA; 2PNDA; 2PNEA; 2POFA; 2PQ7A; 2PR7A; 2PRVA; 2PRXA; 2PU3A; 2PVBA; 2PXXA; 2PYQA; 2Q2FA; 2Q3TA; 2Q5CA; 2Q9KA; 2QCPX; 2QF4A; 2QFEA; 2QGUA; 2QIPA; 2QJLA; 2QJWD; 2QJZB; 2QKVA; 2QL8A; 2QLWA; 2QNGA; 2QNLA; 2QSBA; 2QSKA; 2QSWA; 2QT1A; 2QUDA; 2QZCA; 2R01A; 2R0XA; 2R16A; 2R2ZA; 2R31A; 2R5OA; 2R6UA; 2RA9A; 2RAFB; 2RBDB; 2RE2A; 2RFFA; 2RILA; 2RL8A; 2TNFA; 2TPSA; 2UU8A; 2UV4A; 2V1MA; 2V33A; 2V6VA; 2V76A; 2V7FA; 2V89A; 2V8FA; 2V9VA; 2VB1A; 2VC8A; 2VCLA; 2VH3A; 2VNGA; 2VPTA; 2VQ2A; 2VXTI; 2VY8A; 2VZCA; 2W15A; 2W1JA; 2W1RA; 2W31A; 2W3GA; 2W47A; 2W50A; 2W7AA; 2W7ZA; 2WAGA; 2WCWA; 2WDSA; 2WFIA; 2WFWA; 2WH6A; 2WJ5A; 2WLVA; 2WNKA; 2WNPF; 2WQ4A; 2WQFA; 2WTPA; 2WWEA; 2WWXB; 2WY4A; 2WZOA; 2X32A; 2X3MA; 2X46A; 2X5NA; 2X5YA; 2XETA; 2XHFB; 2XODA; 2XOLA; 2XOMA; 2XQQA; 2XRHA; 2XU3A; 2XW6A; 2Y6XA; 2Y8YA; 2YC3A; 2YH5A; 2YH6A; 2YOGA; 2YZYA; 2Z0JA; 2Z51A; 2Z5WA; 2Z6OA; 2Z98A; 2ZA4B; 2ZCMA; 2ZDPA; 2ZEXA; 2ZFDB; 2ZK9X; 2ZNRA; 2ZOUA; 2ZPMA; 2ZS0C; 2ZW2A; 3A0SA; 3A0YA; 3A2ZA; 3A35A; 3A57A; 3A6RA; 3A8GA; 3A8GB; 3ACHA; 3AGNA; 3AIAA; 3AKBA; 3AKSA; 3B4QA; 3B5MA; 3B5OA; 3B64A; 3B6EA; 3B79A; 3BA3A; 3BEDA; 3BEXA; 3BGUA; 3BHWA; 3BM7A; 3BMZA; 3BO6A; 3BOEA; 3BQPA; 3BRCA; 3BT5A; 3BUUA; 3BWHA; 3BWZA; 3BY8A; 3C8CA; 3C8LB; 3C9AA; 3CBZA; 3CCDA; 3CHMA; 3CI3A; 3CIMA; 3CP7A; 3CT5A; 3CT6A; 3CWRB; 3CZXA; 3D0JA; 3D1PA; 3D3BA; 3D3BJ; 3D4EA; 3D5PA; 3D7JA; 3D9NA; 3D9XA; 3DB7A; 3DKMA; 3DLCA; 3DNJA; 3DO8A; 3DOUA; 3DQPA; 3DQYA; 3DS4A; 3DSBA; 3DWGC; 3DXYA; 3E0XA; 3E4GA; 3E8OB; 3E8TA; 3EA6A; 3EF8A; 3EJVA; 3EO6B; 3EOIA; 3ESSA; 3EUNA; 3EURA; 3EYEA; 3F0DA; 3F14A; 3F2ZA; 3F43A; 3F6VA; 3F7EA; 3F8XD; 3FCNA; 3FDEA; 3FG9A; 3FGVA; 3FGYA; 3FKEA; 3FSAA; 3FSOA; 3FSSA; 3FTDA; 3FYMA; 3FYNA; 3G0KA; 3G16A; 3G7RA; 3G89A; 3GA4A; 3GBWA; 3GE3C; 3GE3E; 3GIUA; 3GKJA; 3GMGA; 3GMXB; 3GNLA; 3GNZP; 3GOCB; 3GOEA; 3GP6A; 3GRDB; 3GWIA; 3GZBF; 3GZRB; 3H0NA; 3H3LB; 3H4OA; 3H5JA; 3H7HA; 3H7HB; 3HF5A; 3HM4B; 3HWUA; 3HX8B; 3HYNA; 3HZPA; 3I7MA; 3I94A; 3IE4A; 3IEZA; 3IISM; 3IMKA; 3IP0A; 3IPJA; 3IQUA; 3IR4A; 3ITFA; 3ITQA; 3IUOA; 3IUWB; 3IV4A; 3IVVA; 3IWFA; 3IX3A; 3IXLA; 3JRVA; 3JTZA; 3JUDA; 3JUMA; 3JXOA; 3K05A; 3K5JA; 3K67A; 3K6YA; 3KE7B; 3KEVA; 3KFFA; 3KGKA; 3KGYB; 3KH1B; 3KKFA; 3KM5A; 3KMHA; 3KORA; 3KU3B; 3KWEA; 3KWRA; 3KYJA; 3KYZA; 3L46A; 3L51B; 3L9AX; 3LAAA; 3LAXA; 3LB2A; 3LD7A; 3LDCA; 3LFKA; 3LHCA; 3LHIA; 3LHNA; 3LLOA; 3LLUA; 3LQBA; 3LW3A; 3LWXA; 3LX3A; 3LYDA; 3LYHB; 3LYPA; 3M0ZA; 3M1XA; 3M3PA; 3M7AB; 3M8JA; 3M9QA; 3MABA; 3MAOA; 3MBRX; 3MC3A; 3MCWB; 3ME7A; 3MEAA; 3MILA; 3MMHA; 3MQZA; 3MR0A; 3MSTA; 3MVCA; 3MVSA; 3MWZA; 3MXNA; 3MXNB; 3MXZA; 3MYXA; 3N01A; 3N08A; 3N10A; 3N6YA; 3NBMA; 3NDHA; 3NEUA; 3NJ2A; 3NJNA; 3NKEA; 3NO7A; 3NOHA; 3NPDA; 3NR5A; 3NUFA; 3NZLA; 3NZMA; 3O12A; 3O2RA; 3O7BA; 3OBLA; 3OBQA; 3OE3A; 3OFGA; 3OGNA; 3OHEA; 3OMDA; 3ON9A; 3OOUA; 3ORUA; 3OV5A; 3OV9A; 3OXPA; 3P4HA; 3PD7A; 3PESA; 3PIWA; 3PJPA; 3PLUA; 3PLWA; 3PMCA; 3PN3B; 3POJA; 3PP2A; 3PUCA; 3PVHA; 3PVIA; 3Q46A; 3Q4OA; 3Q64A; 3Q6BA; 3Q7RA; 3QB8A; 3QC7A; 3QHBA; 3QL9A; 3QOOA; 3QP4A; 3QPAA; 3QR7A; 3QU3A; 3QU5A; 3QX1A; 3QZBA; 3QZMA; 3QZRA; 3QZXA; 3R2QA; 3R6DA; 3R72A; 3R87A; 3R8JA; 3R9FA; 3RO3A; 3ROBA; 3ROFA; 3RPEA; 3RQ9A; 3RRIA; 3RT2A; 3RWNA; 3RX9A; 3RZNA; 3S0AA; 3S2RA; 3S4EA; 3S6EA; 3S6FA; 3S9XA; 3SEEA; 3SK2A; 3SK7A; 3SO6A; 3SOJA; 3SU6A; 3SUJA; 3SUKA; 3SXMA; 3SY1A; 3T3LA; 3T47A; 3T7LA; 3T90A; 3T92A; 3TG2A; 3TOWA; 3TS3A; 3TT9A; 3TU8A; 3TVJA; 3TYSA; 3U3LC; 3U3ZA; 3U5SA; 3U6GA; 3U7ZA; 3U97A; 3UB6A; 3UFEA; 3UI4A; 3UIDA; 3ULJA; 3ULTA; 3UP3A; 3URRA; 3V46A; 3V4KA; 3VORA; 3VQJA; 3VUBA; 3VVVA; 3VWCA; 3VZ9B; 3W42A; 3WCQA; 3WDNA; 3WH1A; 3WJTA; 3WMVA; 3WQBB; 3WURA; 3WV7A; 3WVAA; 3WZ3A; 3X0TA; 3X2MA; 3X34A; 3ZBDA; 3ZBOB; 3ZFPA; 3ZHIA; 3ZJAA; 3ZN4A; 3ZRXA; 3ZSUA; 3ZVSA; 3ZW5A; 3ZY7A; 3ZYPA; 3ZZOA; 3ZZYA; 4A02A; 4A2VA; 4A4YA; 4A56A; 4A6QA; 4A7UA; 4ACJA; 4AE7A; 4AFFA; 4AFMA; 4AL0A; 4ALZA; 4ANNA; 4AQOA; 4AU1A; 4AVSA; 4AXOA; 4AY0A; 4B0ZA; 4B1MA; 4B5OA; 4B89A; 4B8XA; 4B9GA; 4BFOA; 4BGCA; 4BJIA; 4BK7A; 4BOQA; 4BOUA; 4BPFA; 4BVXA; 4BYZA; 4C6AA; 4CA1A; 4CAYA; 4CE8C; 4CGSA; 4CICA; 4CJ0B; 4CK4A; 4CO8A; 4COGA; 4CUAA; 4CV7A; 4D0QA; 4DB5A; 4DM5A; 4DMIA; 4DQ9A; 4DQJA; 4DRIB; 4DT5A; 4DUQA; 4DYQA; 4E29A; 4E3YA; 4E6FA; 4EA9A; 4EAEA; 4EBGA; 4EFPA; 4EKFA; 4EMNA; 4EP4A; 4EQ8A; 4EQ9A; 4EQAC; 4EQPA; 4ERCA; 4ES1A; 4ESAB; 4EUNA; 4EX6A; 4EZGA; 4F2EA; 4F54A; 4FCHA; 4FN7A; 4FP5D; 4FR9A; 4FTFA; 4FZLA; 4FZPA; 4G0XA; 4G4KA; 4G6TA; 4G6TB; 4G78A; 4G7XA; 4G7XB; 4G9SA; 4G9SB; 4GA2A; 4GB5A; 4GEIA; 4GJZA; 4GMQA; 4GS3A; 4GT8A; 4GT9A; 4GUCA; 4GWBA; 4GZCA; 4H3UA; 4H6CI; 4H7WA; 4HBQA; 4HBZA; 4HC9A; 4HCJA; 4HCSA; 4HE6A; 4HFSA; 4HJIA; 4HLYA; 4HMSA; 4HQZA; 4HS2A; 4HWMA; 4HY4A; 4I0WA; 4I1KA; 4I4OA; 4I66A; 4I6RA; 4I6XA; 4I8HA; 4IAUA; 4IEJA; 4IKDA; 4IL7A; 4INWA; 4IPUA; 4IUMA; 4IX7A; 4J42A; 4J5RA; 4J8SA; 4J9YB; 4JDUA; 4JEMA; 4JF8A; 4JG2A; 4JGIA; 4JGLA; 4JHTA; 4JK8A; 4JM1A; 4JQFA; 4JTMA; 4JXRA; 4JZ5A; 4K12B; 4K5AA; 4K7BA; 4K82A; 4KDWA; 4KEFA; 4KM6A; 4KQDA; 4KQPA; 4KT3A; 4KT3B; 4KU0A; 4KU0D; 4L8PA; 4L9NA; 4LA2A; 4LD1A; 4LJOA; 4LLDB; 4LLYA; 4LPQA; 4LRUA; 4LTTA; 4LUAA; 4LUPA; 4M1GA; 4M1XA; 4M91A; 4M9KA; 4MAIA; 4MAKA; 4MAQA; 4MAXA; 4MJDA; 4MNOA; 4MTMA; 4MTUA; 4MUVA; 4MXTA; 4MZJA; 4N2PA; 4N30A; 4NBPA; 4NDSA; 4NG0A; 4NI6A; 4NKPA; 4NN2A; 4NOAA; 4NOHA; 4NXYA; 4NYQA; 4O06A; 4O0AA; 4O6UA; 4OA3A; 4OD6A; 4OE9A; 4OHJA; 4OI3A; 4ONRA; 4OQ9A; 4P0TA; 4P3HA; 4P3VA; 4P5EA; 4P82A; 4PDNA; 4PH2A; 4PH8A; 4PHJA; 4PI8A; 4PJ2A; 4PQDA; 4PQHA; 4PS6A; 4PSFA; 4PSSA; 4PWOA; 4PWWA; 4PXYA; 4PZ3A; 4Q29A; 4Q2SA; 4Q4W3; 4Q53A; 4Q7OA; 4QA8A; 4QASA; 4QBOA; 4QC6A; 4QDJA; 4QHQA; 4QI3B; 4QKDA; 4QLPA; 4QLPB; 4QM6A; 4QP5A; 4QPNA; 4QPWA; 4QXLA; 4QXVA; 4QY7A; 4R03A; 4R1JA; 4R81A; 4R9PA; 4RAYA; 4REIA; 4REOA; 4RFUA; 4RLCA; 4RP3A; 4RPTA; 4RRIA; 4RTHA; 4RU3A; 4RUQA; 4RVQA; 4RWUA; 4RWWA; 4RXVA; 4RYOA; 4RZ9A; 4S1PA; 4TKCA; 4TNFA; 4TPVA; 4TQXA; 4TSDB; 4TTWA; 4TXRA; 4TYZA; 4U5HA; 4U5RA; 4UE0A; 4UE8A; 4UFQA; 4UHQA; 4UHTA; 4UJ7A; 4UQWA; 4UQZB; 4UTUA; 4UU3B; 4UWWA; 4UYRA; 4V1GA; 4V1JA; 4V1KA; 4V4ME; 4W64A; 4W6YA; 4W78C; 4W78H; 4W79A; 4W8HA; 4W8QA; 4W9ZA; 4WBJA; 4WDCA; 4WF5A; 4WH9A; 4WHSD; 4WJTA; 4WN5A; 4WPKA; 4WPYA; 4WQKA; 4WRIA; 4WSFA; 4WUIA; 4WWFA; 4WZXA; 4X2RA; 4X5PA; 4X84A; 4X9RA; 4XB4A; 4XBAB; 4XEDA; 4XEZA; 4XINA; 4XPXA; 4XTBA; 4XXLA; 4XZFA; 4Y1WA; 4Y2FA; 4Y7LA; 4Y88A; 4Y9IA; 4YCBA; 4YE7A; 4YECA; 4YECB; 4YEPA; 4YMYA; 4YORA; 4YSIA; 4YTDA; 4YTKA; 4YTWC; 4YTWD; 4YUDA; 4YWAA; 4YWKA; 4YZ0A; 4Z39A; 4Z3GA; 4Z47A; 4Z7XA; 4Z9HA; 4ZAVA; 4ZBGA; 4ZBHA; 4ZEYA; 4ZGFA; 4ZILA; 4ZJHA; 4ZLDA; 4ZQXA; 4ZV0A; 4ZV0B; 4ZV5A; 4ZVCA; 4ZVFA; 4ZZ1A; 5A0LB; 5A0NA; 5A0YC; 5A67A; 5A99A; 5AE0A; 5AFWA; 5AGRA; 5AIGA; 5AIMA; 5ANVA; 5AOTA; 5AOZA; 5APGA; 5AZWA; 5AZXA; 5B08A; 5B1AD; 5B1AE; 5B1AG; 5B1AH; 5B4BA; 5B6CA; 5B7HA; 5B8DA; 5BMTA; 5BOBA; 5BS1A; 5BTYA; 5BY5A; 5BY8A; 5BY8B; 5C17A; 5C2UA; 5C33B; 5C5ZA; 5CDKA; 5CEGB; 5CKLA; 5CL8A; 5COFA; 5COWA; 5CPHA; 5CR4A; 5CSDA; 5CTMA; 5CTVA; 5CUOA; 5CVWA; 5CWGA; 5CWHA; 5CWLA; 5D66A; 5DBLA; 5DGJA; 5DHDA; 5DICA; 5DLEA; 5DMDA; 5DZEA; 5E1WA; 5E4GA; 5E9PA;

5EDFA; 5EL3A; 5EL9A; 5EMIA; 5EPWA; 5EU0A; 5EW0A; 5EWOA; 5EWYA; 5EZUA; 5F47A; 5F4CA; 5F6RA; 5FAFA; 5FEBA; 5FISA; 5FJDA; 5FJLA; 5FMUA; 5FPZA; 5FU5A; 5G38A; 5G3YA; 5G51A; 5GGNA; 5GI7A; 5GNFA; 5GNGA; 5GQIA; 5GRQA; 5GTUA; 5GV0A; 5H0MA; 5H0QA; 5H3VA; 5H6XA; 5H9NA; 5HB6A; 5HB7A; 5HBPA; 5HDKA; 5HHED; 5HJ1A; 5HOEA; 5HQHA; 5HRAA; 5HTLA; 5HUBA; 5HWKA; 5I0YA; 5I45A; 5I5NA; 5IDBB; 5IHFA; 5II6A; 5IMAA; 5IO9A; 5ISVA; 5IUCB; 5IWHA; 5IXBA; 5IXHA; 5J1NA; 5J1SB; 5J3TA; 5J4FA; 5J4LA; 5J4OA; 5J6YA; 5JBNA; 5JDKA; 5JE2B; 5JELA; 5JIGA; 5JJ2A; 5JPHA; 5JUGA; 5JUHA; 5K34A; 5K8JA; 5K91A; 5KLEA; 5KNHI; 5KP7B; 5KVBA; 5KVGE; 5L37C; 5L74A; 5LALA; 5LB7A; 5LBDA; 5LEOA; 5LHMA; 5LHXA; 5LJMA; 5LJPA; 5LNDA; 5LP9A; 5LQ6A; 5LS4A; 5LS7B; 5LS7D; 5LT5A; 5LY8A; 5LZKA; 5LZNA; 5M0WA; 5M0YB; 5M1MA; 5M1PA; 5M2OB; 5M2PA; 5M33A; 5M72A; 5MAWE; 5MDUA; 5MFOA; 5MJRA; 5ML3B; 5MPWA; 5MR1A; 5MSZA; 5MY7A; 5N86A; 5N8AX; 5NCGA; 5NHUI; 5NJOA; 5NQOA; 5NR4B; 5NRMA; 5NSAA; 5NT7A; 5NWPA; 5NZOB; 5O2XA; 5O45A; 5O63A; 5O9MA; 5OBTA; 5OD4A; 5OF1A; 5OHQA; 5OJCA; 5OK6A; 5OL4A; 5OL4B; 5OL9A; 5OMTA; 5ONKA; 5OPZA; 5OXZA; 5P9VA; 5QHHA; 5QI0A; 5QIVA; 5QOQA; 5QS9A; 5SV5A; 5SZCA; 5T7AA; 5TIFA; 5TJZA; 5TKWA; 5TPIA; 5UEBA; 5UFYA; 5UMRA; 5UOUA; 5UUKA; 5UWZA; 5UX1A; 5UZGB; 5V01A; 5V1VB; 5V1YB; 5V3NA; 5V5HA; 5V6JA; 5VBDA; 5VGBA; 5VGBB; 5VGLA; 5VHGA; 5VJTA; 5VX5A; 5VXVA; 5W0GA; 5W2FA; 5W4AC; 5WD9A; 5WECA; 5WFYA; 5WJPA; 5WK0A; 5WKRA; 5WSFA; 5WUCA; 5X57A; 5X5MA; 5X7LA; 5X9LA; 5XBCA; 5XBIA; 5XDHC; 5XJ5A; 5XK6A; 5XLUB; 5XM5B; 5XN3A; 5XVJA; 5XZ4A; 5Y4TA; 5YA6A; 5YDDA; 5YDEA; 5YDNA; 5YGBA; 5YH4A; 5YHRA; 5YKZA; 5YNXB; 5YQJA; 5YRVB; 5YRVC; 5YSIA; 5YUGA; 5YVKA; 5YWRB; 5YXMA; 5YZPA; 5Z0DB; 5Z42A; 5Z51A; 5Z6DA; 5ZBYA; 5ZCYA; 5ZDMA; 5ZGXA; 5ZHOA; 5ZIMA; 5ZKEB; 5ZO3A; 5ZOHA; 5ZRYA; 5ZT3A; 5ZZAP; 6A02A; 6A2QA; 6A56A; 6A58A; 6A5DB; 6A80B; 6AC0A; 6AISO; 6AJPA; 6AJZA; 6AM3X; 6ANZA; 6AO9A; 6AT6A; 6AVXA; 6B1KA; 6B8FA; 6B9HA; 6B9XA; 6B9XB; 6B9XC; 6B9XE; 6BCBA; 6BCDA; 6BHDA; 6BLMA; 6BO0A; 6BTDA; 6BXDA; 6BXGA; 6C1XA; 6C8CB; 6CB7A; 6CBRA; 6CBUA; 6CD9A; 6CHXA; 6CN8A; 6CNWA; 6CPBB; 6CVAA; 6CWMA; 6D0HA; 6D9NA; 6DCEA; 6DCJA; 6DCMA; 6DGAA; 6DGGA; 6DKQA; 6DNMA; 6DOPA; 6DTVA; 6DUBA; 6E1FB; 6E3AA; 6E55D; 6E5FA; 6E5XA; 6E7EA; 6EDVA; 6EHIA; 6EIOA; 6ELMA; 6ELVA; 6ENIA; 6ER1A; 6ER4A; 6ER6A; 6ETLA; 6EVNA; 6EWHA; 6EWLA; 6EWMA; 6EXMA; 6F5CA; 6FBQA; 6FC0B; 6FDGA; 6FEAC; 6FFAA; 6FG8B; 6FJ7A; 6FJNA; 6FLKA; 6FM5A; 6FMBA; 6FPQA; 6FTOC; 6FU9A; 6FU9B; 6FVIA; 6FXDA; 6G1CV; 6G1IA; 6G25A; 6G6KA; 6G8YA; 6G96A; 6GAJA; 6GBIA; 6GCFA; 6GCVA; 6GDJA; 6GDXA; 6GG1A; 6GI4B; 6GKSA; 6GKXA; 6GN5A; 6GQZA; 6GREA; 6GS6A; 6GV3A; 6GV5A; 6GV8A; 6GZ0A; 6GZ8A; 6H24A; 6H40A; 6H8GA; 6H8OA; 6H96A; 6H9YA; 6HERA; 6HFMA; 6HHNA; 6HIUB; 6HOAA; 6HQCA; 6HRNA; 6HS0A; 6HSAA; 6HX0A; 6HYYA; 6I0IA; 6I4DG; 6I4EG; 6I5OD; 6I65A; 6I6SA; 6I8YA; 6ICSO; 6IGGA; 6IJEB; 6IQCA; 6ITAA; 6IX1B; 6IY4I; 6J0EB; 6J4DA; 6J6PA; 6J93A; 6JGJA; 6JJTD; 6JK4A; 6JLEA; 6JM5B; 6JNYB; 6JPTA; 6JSAA; 6K39A; 6KBXB; 6KL1A; 6KO8A; 6KWZA; 6LACA; 6LG2B; 6M9MA; 6MASH; 6MDHA; 6MDWA; 6MICA; 6ML4A; 6MM2A; 6MU0A; 6MX3A; 6MYID; 6N6AA; 6N6JA; 6N8YA; 6N9HA; 6NDTB; 6NE2A; 6NFRA; 6NK0B; 6NLQC; 6NPPA; 6NSVA; 6NUHA; 6NVXA; 6NX5C; 6NZTA; 6O2VA; 6O54X; 6OHKA; 6OSXA; 6OVIC; 6OW7Q; 6P28A; 6P29B; 6PJVA; 6PYMA; 6Q10A; 6Q1HG; 6Q1MA; 6Q58A; 6QAZA; 6QFQA; 6QGGA; 6QHGA; 6QJAA; 6QLHA; 6QLLA; 6QQHA; 6QSPB; 6QU6A; 6QVFD; 6QVHA; 6QW0B; 6QYBA; 6R01A; 6R1DB; 6R1GB; 6R2WL; 6R3MA; 6R4ZA; 6R54B; 6R55A; 6R5AB; 6RFKS; 6RK0A; 6RNIA; 6RRVA; 6RT6C; 6RWTA; 6S2MA; 6S5WB; 6SIDA; 6SJAB; 6SLLB; 6SOOA; 6SVLF; 6T3XA; 6T84A; 6TGSA; 6TYJA; 6TYUA; 6TZXA; 6U54B; 6U66A; 6UOFA; 6UXEB; 6UXED; 6UYRA
